# Material Legacies and Environmental Constraints Underlie Fire Resilience of a Dominant Boreal Forest Type

**DOI:** 10.1007/s10021-022-00772-7

**Published:** 2022-06-29

**Authors:** Nicola J. Day, Jill F. Johnstone, Kirsten A. Reid, Steven G. Cumming, Michelle C. Mack, Merritt R. Turetsky, Xanthe J. Walker, Jennifer L. Baltzer

**Affiliations:** 1 Biology Department, Wilfrid Laurier University, Waterloo, Ontario Canada; 2 School of Biological Sciences, Victoria University of Wellington, Wellington, New Zealand; 3 YukonU Research Centre, Yukon University, Whitehorse, Yukon Canada; 4 Institute of Arctic Biology, University of Alaska Fairbanks, Fairbanks, Alaska USA; 5 Faculté de foresterie, de géographie et de géomatique, Département des sciences du bois et de la forêt, Université Laval, Québec, Québec Canada; 6 Center for Ecosystem Science and Society, Northern Arizona University, Flagstaff, Arizona USA; 7 Institute of Arctic and Alpine Research, University of Colorado Boulder, Boulder, Colorado USA; 8 Present Address: Department of Geography, Memorial University, St. John’s, Newfoundland and Labrador Canada

**Keywords:** Boreal forest, Drought, *Pinus banksiana*, *Populus tremuloides*, Seed limitation, Seedbed, Taiga plains, Taiga shield, Vegetation change, Wildfire

## Abstract

Resilience of plant communities to disturbance is supported by multiple mechanisms, including ecological legacies affecting propagule availability, species’ environmental tolerances, and biotic interactions. Understanding the relative importance of these mechanisms for plant community resilience supports predictions of where and how resilience will be altered with disturbance. We tested mechanisms underlying resilience of forests dominated by black spruce (*Picea mariana*) to fire disturbance across a heterogeneous forest landscape in the Northwest Territories, Canada. We combined surveys of naturally regenerating seedlings at 219 burned plots with experimental manipulations of ecological legacies via seed addition of four tree species and vertebrate exclosures to limit granivory and herbivory at 30 plots varying in moisture and fire severity. Black spruce recovery was greatest where it dominated pre-fire, at wet sites with deep residual soil organic layers, and fire conditions of low soil or canopy combustion and longer return intervals. Experimental addition of seed indicated all species were seed-limited, emphasizing the importance of propagule legacies. Black spruce and birch (*Betula papyrifera*) recruitment were enhanced with vertebrate exclusion. Our combination of observational and experimental studies demonstrates black spruce is vulnerable to effects of increased fire activity that erode ecological legacies. Moreover, black spruce relies on wet areas with deep soil organic layers where other species are less competitive. However, other species can colonize these areas if enough seed is available or soil moisture is altered by climate change. Testing mechanisms underlying species’ resilience to disturbance aids predictions of where vegetation will transform with effects of climate change.

**Supplementary Information:**

The online version contains supplementary material available at 10.1007/s10021-022-00772-7.

## Introduction

Large, high intensity fires are becoming more frequent and exceeding historical norms due to climate change causing drought and extreme fire weather in many areas globally (Bowman and others 2020). Changes in fire activity can impact forest resilience by reducing its ability to return to pre-disturbance vegetation conditions (Turner 2010; Johnstone and others 2016; Nolan and others 2021). Fire-driven losses or shifts in dominant vegetation is occurring in many regions, including eucalyptus forests in southern Australia (Bowman and others 2016), lodgepole pine forests in interior USA (Turner and others 2019), and boreal forests in eastern and western North America (Boiffin and Munson 2013; Whitman and others 2019). Shifts in dominant vegetation can influence functional capabilities of the ecosystem, such as ability to store carbon or provide habitat for wildlife. Knowing recovery patterns of dominant vegetation to fire will enhance our ability to predict when and where resilience will be lost and potential impacts on ecosystem properties.

Resilience of dominant vegetation to disturbances, such as fire, is often maintained by ecological memory in the form of legacies (Franklin and others 2000; Johnstone and others 2016). Disturbance characteristics interact with pre-disturbance vegetation and environmental conditions to determine the transmission of ecological memory via material or information legacies that shape ecosystem recovery (Johnstone and others 2016, Turner and others 2019). Material legacies of organisms, propagules, and other biological materials are impacted by disturbance characteristics, such as severity, frequency, and size, with implications for recovery through availability of seeds and seedbeds for regeneration, for example. Information legacies are inherited traits or adaptations that shape species tolerances of environmental conditions and disturbance regimes and affect the suite of conditions that can support species recovery. Disturbance characteristics that lie outside the historic range of variability, such as unusually severe or frequent fires, can disrupt the transmission of key material or information legacies that support resilience, leading to shifts in dominant vegetation or ecological states (Johnstone and others 2016). Biotic interactions, such as granivory or herbivory, can further alter the transmission or effects of material and information legacies and can themselves also be modified by disturbance or other environmental change (Frei and others 2018).

In the North American boreal forest, black spruce (*Picea mariana* (Mill) BSP) stands represent a dominant stand type that has been resilient to repeated fire disturbance for millennia (MacDonald 2000; Girardin and others 2013; Kelly and others 2013). Black spruce is considered a fire-adapted species, with aerial seedbanks held within semi-serotinous cones and accumulation of ladder fuels that promote flammability (Greene and others 1999; Cumming 2001). Nevertheless, widespread losses of black spruce are occurring after wildfires across the North American boreal forest, particularly in areas with greater fire severity, shallower soil organic layers (SOL), and warm and dry post-fire conditions (Baltzer and others 2021). Thus, we need to better understand the mechanisms underlying black spruce resilience, particularly the conditions that can lead to a decline in spruce recovery and concomitant shifts to alternative species composition or dominance. Specifically, we need to tease apart sensitivity of black spruce to factors that are typically confounded in observational studies, such as simultaneous changes in fire severity and frequency that alter both seed availability and seedbed conditions. Similarly, granivory or herbivory of mammals can be sufficient to modify patterns of seedling recruitment (Côté and others 2003; Olnes and Kielland 2017; Olnes and others 2017). Granivores and herbivores, such as small mammals and snowshoe hares (*Lepus americanus*), can be abundant in boreal forests even after extreme fire (Zwolak and others 2012). However, their role in seedling recruitment in boreal forests remains poorly quantified (Evans and Brown 2017; Frei and others 2018; Peters and others 2004) and greater exploration on how they interact with legacies to mediate ecological memory is needed. This requires experimental studies coupled with observational studies to assess consistency of patterns in different conditions and to disentangle confounding factors on regeneration of seedlings.

Black spruce is ideal as a case study to fill knowledge gaps regarding the relative importance of vegetation legacies, abiotic conditions, and biotic interactions for resilience to fire under continued climate change. Direct regeneration, where post-disturbance plant community composition is restored to that of pre-disturbance within a short period of time, is the dominant mode of post-fire succession in western North American boreal forests (Ilisson and Chen 2009). Therefore, species dominance of regenerating seedlings in the years immediately following fire is strongly correlated with species dominance later in succession (Gutsell and Johnson 2002; Johnstone and others 2004, 2020; Shenoy and others 2011). Black spruce requires at least 50 years to build viable seed banks to support recruitment (Brown and Johnstone 2012; Viglas and others 2013; Whitman and others 2019). Thus, black spruce may experience increased sexual immaturity risk as fire return intervals shorten, leading to relative declines in recruitment due to losses of material legacies (also known as ‘interval squeeze’; Enright and others 2015; Nolan and others 2021). Seed-addition studies that remove material legacy limitations in the form of propagules show that black spruce can establish in deep SOL, which is prohibitive to the establishment of other species (Charron and Greene 2002; Johnstone and Chapin 2006; Brown and others 2015). This demonstrates the role of information legacies in the form of species traits that affect the realized niche for recruitment of trees in the boreal forest. Conifers such as black spruce and jack pine (*Pinus banksiana* Lamb*.*) rely on aerial seedbanks in serotinous or semi-serotinous cones to regenerate after fire, while small-seeded broadleaf species such as birch (*Betula papyrifera* Marsh.) and aspen (*Populus tremuloides* Michx.) disperse into a site after fire (Johnstone and Chapin 2006; Greene and others 2007). Under high fire severity or frequency, the SOL can be entirely combusted to expose mineral soil seedbeds that improve access to moisture and nutrients and favor faster-growing species such as aspen or jack pine over more conservative species such as black spruce (Greene and others 2007; Johnstone and others 2010a; Brown and others 2015; Whitman and others 2019). Within the conifers, jack pine may be positively impacted by intense fire activity relative to black spruce because it can produce seeds at a younger age and its cones are better able to withstand high severity fires (Burns and Honkala 1990; Lavoie and Sirois 1998; Greene and others 2004).

Here, we aim to understand patterns and mechanisms of ecosystem resilience to fire across a heterogeneous forest landscape within two major ecozones in Canada’s western boreal forest after an extreme fire year culminating from a multi-year drought and extreme lightning activity (Kochtubajda and others 2019). We combined observational and experimental approaches to understand the relative importance of three mechanisms hypothesized to underlie resilience of black spruce forests to fire: (a) material legacies in the form of seed availability and seedbed conditions, (b) information legacies affecting regeneration strategy and tolerance of environmental conditions, and (c) biotic effects of vertebrate granivory or herbivory. We surveyed natural tree seedling regeneration across 219 plots in conifer-dominated stands that encompassed a range of environmental conditions, fire history, and fire severity. At these sites, recovery of pre-fire canopy composition to a similar post-fire tree seedling composition indicates high resilience of these forests to disturbance. At a subset of 30 sites, we experimentally added seed to assess the relative importance of material legacies in the form of propagules and seedbed conditions for seedling recruitment of black spruce, jack pine, aspen, and birch. An exclosure treatment at each site allowed us to assess the impacts of vertebrate granivory or herbivory on seedling densities. We hypothesized that conifers would be most resilient under low fire severity and canopy combustion, and longer time between fires. However, jack pine recruitment may be favored as time between fires shortens (because they mature faster) and under higher canopy combustion (because their cones are fully serotinous). Compositional shifts to broadleaf taxa would be in greater densities under high fire severity and mineral soil cover. In the absence of seed limitation, we hypothesized there would be more seedlings of all species with greater mineral soil cover and shallow residual SOL because they are superior seedbeds, but these effects would be greater for broadleaf species. For all species, we expected seedling densities would increase with the exclusion of vertebrates. By combining the two study types at the same sites and across a suite of environmental conditions, we bring new understanding of the relative importance of different mechanisms to forest recovery after fire, and thus aid predictions in where resilience is most likely to be lost in future wildfires.

## Methods

### Study Areas

Our study took place in the years following an extreme fire event attributed to a prolonged, multi-year drought where 2.85 Mha of boreal forest burned in the northwest territories (NWT), Canada in 2014 (Canadian Interagency Forest Fire Centre 2014; Walker and others 2018b; Kochtubajda and others 2019). This fire event is unprecedented in the NWT’s fire history, burning an area more than eight times greater than the annual mean. Our study encompasses burned areas in two of the dominant parent material types in the North American boreal forest, represented by the Taiga Plains (hereafter Plains) and Taiga Shield ecozones (hereafter Shield), which meet in the NWT. Thus, our sites experience broadly similar climatic conditions but are underlain by different soil properties. The Plains is a mix of undulating glacial till and peatlands with permafrost in wetter areas (Ecosystem Classification Group 2009). The Shield, in the eastern part of the NWT, has hilly pre-Cambrian bedrock with thin till, overlain in places by layers of clay, sand, and gravel (Ecosystem Classification Group 2008). Both ecozones are characterized by open, slow growing forest dominated by black spruce and/or jack pine. In dry areas, aspen can be found on the Plains and paper birch on the Shield. Tamarack (*Larix laricina* (Du Roi) K. Koch) and white spruce (*Picea glauca* (Moench) Voss) are also present in some locations. These are the full suite of trees that are dominant in the North American boreal region, which represent a large diversity of evolutionary adaptations (information legacies; Johnstone and others 2016) for post-fire recruitment: semi-serotinous black spruce, fully serotinous jack pine, and small-seeded dispersers aspen and birch. Moreover, we have the dry climate of the western North American interior with crown-replacing fires occurring approximately every 100 years (Larsen 1997), with the presence of jack pine that is typical of eastern forests but absent from Alaska. All sample locations were within the discontinuous permafrost zone (Zhang and others 1999) and site soil conditions included both seasonally frozen soils or those with deep or near-surface permafrost. Mean annual temperatures measured for 1981–2010 are − 2.5 °C in Hay River in the Plains (60.82°N, − 115.79°W) and − 4.3 °C in Yellowknife in the Shield (62.45°N, − 114.37°W), with a mean annual precipitation of 336 mm and 228 mm, respectively (Environment and Climate Change Canada 2018).

### Natural Seedling Regeneration 2–4 Years After Fire

During June–August 2015–16, 219 plots were established in seven of the 2014 burn scars (Figure S1): 133 plots in the Plains and 86 in the Shield. Plots were located using a stratified random design to sample forest stands dominated by conifers before fire. Within each burn scar, we identified pre-fire strata of medium, low, and sparse conifer density using the Land Cover Classification of Canada 2005 (Latifovic and others 2008). Random points within strata were constrained to within 1 km of roads or lakeshores for access. On the ground, each randomly-generated point was assigned to one of six moisture classes based on site and soil drainage conditions from xeric (driest) to subhygric (wettest; Johnstone and others 2008). To capture soil moisture conditions across the landscape, we co-located at least one, but usually two plots of a different moisture class within 500 m of each randomly selected plot (see Walker and others 2018a for more information about study design).

Each plot comprised two parallel 30 m transects running north spaced 2 m apart (60 m^2^). To assess post-fire seedling regeneration, five 1 × 1 m quadrats were established at 6 m intervals along the eastern transect and tree seedlings of each species were identified and counted; thus, quadrats were nested within plots. We use data from seedling counts that occurred between June and August in 2016–18 (2–4 years after fire) because of difficulties in determining species-level identities of conifer seedlings in the year immediately following fire. There was 100% mortality of trees at most plots, making it easy to determine seedlings that had germinated after fire. Some individuals of aspen likely resprouted from rhizomes/suckers, although the majority were from seed (Day and others 2020). We measured variables indicative of seedbed conditions. Within each quadrat, we estimated percent cover of exposed mineral soil. Adjacent to each quadrat, we measured residual soil organic layer (rSOL) thickness using soil pits or frost probing to estimate depth to mineral soil. Burn depth and proportion SOL combusted were estimated using measurements in the 2014 burned plots combined with calibrations from mature plots with no record of burning (prior to 1965; for details and data see Walker and others 2018a, 2018b). rSOL depth was correlated with moisture category (Figure S2) and our fire severity metric of proportion SOL combusted (*r* =  − 0.71, *t* =  − 14.73, *P* < 0.05).

We identified and counted every tree in the 60 m^2^ plot area to assess pre-fire stand composition, including fallen trees killed by fire. Each tree was assigned a combustion category between 0 (alive, no combustion) and 3 (high combustion with only trunk and large branches remaining). Canopy tree ages can provide good estimates of time since previous fire in these stands because rapid germination of tree seedlings in the initial years following fire results in even-aged cohorts in these forests (Greene and others 1999). We collected basal tree disks or cores from five trees of each dominant conifer species representing the prevailing size class in each plot and counted their rings (Walker and others 2018a). Samples were sanded and scanned and ages determined using Cybis CooRecorder v.7.8 (Larsson 2006) or WinDendro 2009 (Regent Instruments Canada Inc. 2009). Stand age for each plot was calculated based on recruitment cohorts (generally ± 20 years; see Walker and others 2018a for details). Mean stand age (time since fire) was 102 years (± 46 years; Table S1).

### Seed Addition Experiment

We conducted an experiment to understand conditions that promoted regeneration of different tree species in the absence of vegetation legacies that impose seed limitation. Plots (*n* = 30) were selected from the broader dataset in the Plains to span a range of conditions in pre-fire stand composition (jack pine or black spruce dominance), site moisture class, and rSOL depth (Table S1). This experiment was only conducted on the Plains because there was greater variation in pre- and post-fire conditions and more plots were accessible by road on this ecozone. The seed addition experiment was established 5–10 m east of the main plot along a parallel 30 m transect in June 2016, two years post-fire. Five blocks (13/30 plots) or six blocks (17/30 plots) were marked at regular 6 m intervals for a total of 168 1 × 1.5 m blocks. Within each plot, the southern block was covered with a wire mesh exclosure (gauge 6.4–8.5 mm) and secured to the ground with pegs (Figure S3). Exclosures were designed to exclude vertebrate granivores, such as voles (*Microtus* sp. and *Myodes* sp.) and deer mice (*Peromyscus maniculatus*), and larger herbivores, such as snowshoe hares (*Lepus americanus*). The exclosures also likely deterred larger vertebrate herbivores and prevented granivory by birds. Each block comprised six subplots (50 cm × 50 cm) used for seed treatments. We recorded percent cover of mineral soil in each subplot and excavated a soil core to measure rSOL depth in one subplot per block designated for destructive sampling.

Seeds of black spruce, jack pine, birch, and aspen were collected at sites within the Plains during 2015 (Table S2). Aspen seed was supplemented with seeds from Lac La Ronge in northern Saskatchewan collected in June 2016. Seeds were stored in airtight containers at 4 °C. Seed addition treatments were randomly assigned to five of the six subplots within blocks: jack pine, black spruce, aspen, birch, or control (not seeded). We aimed to add enough seeds to saturate microsites available for seedlings to germinate while accounting for viability (Table S2). Seeding coincided with the natural release of aspen seed (early summer). Black spruce and jack pine seeds were cold-wet stratified for three weeks prior to seeding in early summer. Jack pine was only added at 26 plots (146 subplots) due to some seeds being compromised by mold. Unstratified birch seeds were seeded in late summer (end of August to early September), which though about 1–2 months earlier than their natural release was the latest logistically feasible date. We present results from seedling counts where all individuals were identified in all subplots at the 30 sites in 2018 (4 years post-fire, 3 years post-seeding).

### Data Analyses

All data analyses were conducted in R v.3.6.0 (R Core Development Team 2019) with packages tidyverse (Wickham 2017), vegan (Oksanen and others 2019), glmmTMB (Brooks and others 2017), DHARMa (Hartig 2019), egg (Auguie 2019), and ggeffects (Lüdecke 2018). *R* code is provided as supplementary material. Predictors were centered and standardized in all regression models and assumptions were checked. Data from the plains and shield were analyzed separately to account for the variation in responses and different broadleaf species between the two ecozones.

#### What are the Conditions that Promote Black Spruce Resilience?

We first assessed changes in dominance of each canopy species pre- and post-fire, where dominance was assigned as species with > 50% stems. Few plots did not exhibit clear dominance of one particular species (Plains: 15/133 plots, Shield: 6/86 plots). We then used ordination to assess the direction and degree of shifts in canopy composition from pre- to post-fire, and modeled their drivers based on pre- and post-fire compositional trajectories. For each ecozone, principal co-ordinates analysis (PCoA) were performed specifying a Bray–Curtis dissimilarity matrix (Legendre and Legendre 2012). The multivariate observations were pre- and post-fire proportions of stems or seedlings of canopy species for each plot (species densities standardized by total plot density). Species included were black spruce, jack pine, birch, aspen, tamarack, and white spruce. Burned pre-fire stems that were not identifiable to genus or species were omitted and plots with zero seedlings of any species were omitted because dissimilarities cannot be calculated with empty sites (resulting matrices: plains 120 plots: dimensions 240 plot-times × 7 species; shield 79 plots: dimensions 158 × 7).

**Figure 1:**
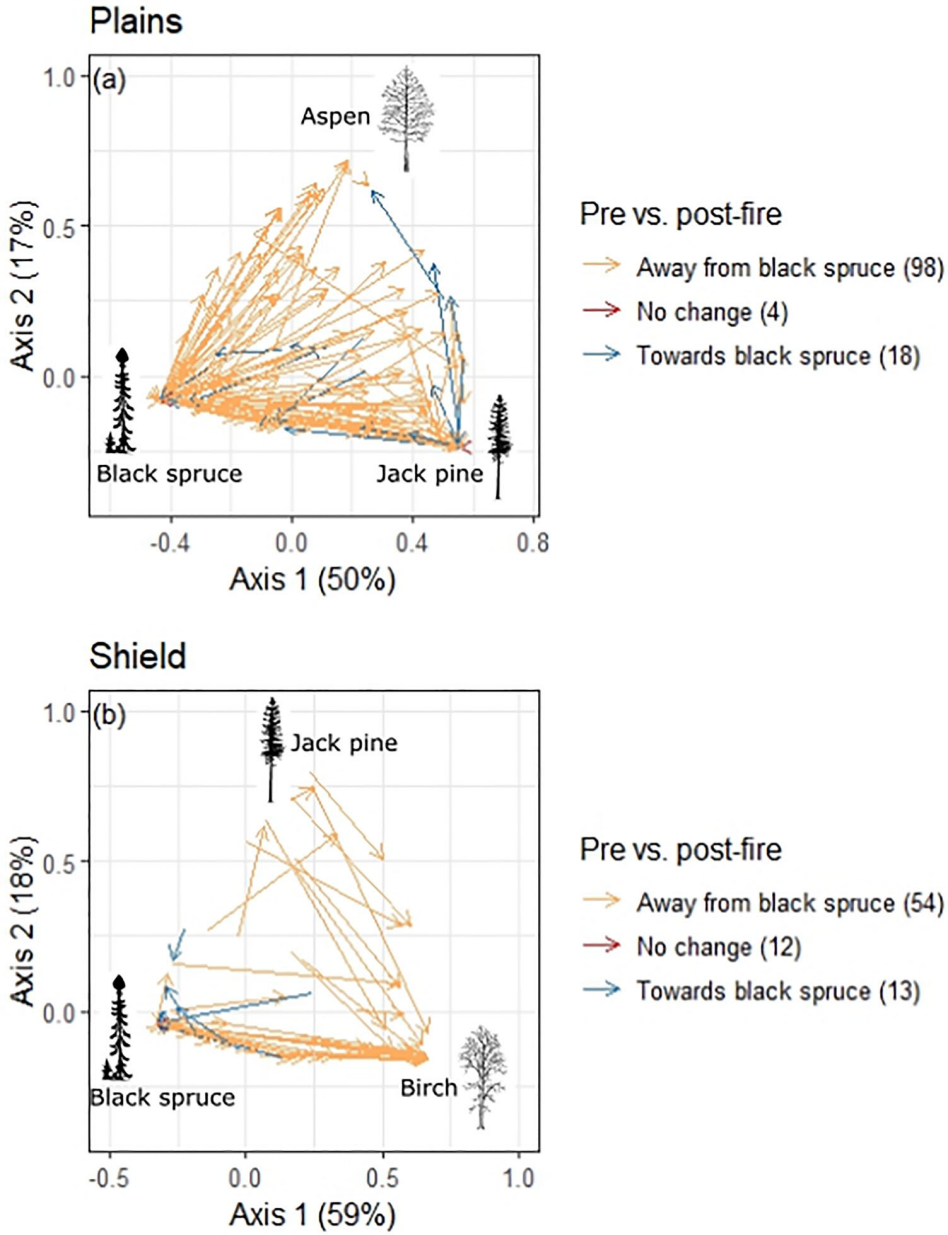
Shifts in tree species composition under natural regeneration (no seed addition): Site scores from principal co-ordinates analysis (PCoA) ordination on pairwise Bray–Curtis distance for forest plots in the, **a** Taiga Plains (7 species and 120 plots) and **b** Taiga Shield (7 species and 79 plots) of the Northwest Territories, Canada. Arrows denote the compositional change in each plot, connecting pre-fire to post-fire site scores. Values on the axis labels indicate percentage of total variation explained by each PCoA axis. Values in brackets in the legend denote how many plots were in each category. Tree images show approximate locations of tree species centroids in ordination space (see Figure S4).

We modeled drivers of the direction of plot compositional shifts along the first two PCoA axes, representing the main axis of variation in composition (see Results: Figures 1 and S4). The first axis in the Plains showed a gradient from black spruce to jack pine and the first axis in the Shield showed a gradient from conifer to birch dominance. Therefore, the difference between the pre-fire and post-fire axis 1 score for each plot represented the shift away from black spruce in both ecozones, where small differences indicated small changes in composition and suggests resilience. Conversely, large distances indicated large shifts in species composition away from pre-fire canopy composition. Differences between the pre- and post-fire axis 1 scores were modeled (post–pre) by linear regression for each ecozone. The predictors were mean plot mineral soil cover, mean rSOL depth, mean canopy combustion, and stand age. Pre-fire PCoA axis 1 scores were also included, as indicators of pre-fire composition.

We also ran generalized linear models with a binomial response to assess the proportion of seedlings that were black spruce, relative to the total number of seedlings at a plot. Only plots that had black spruce seedlings were included in the analyses (Plains: *n* = 91; Shield: *n* = 77). The predictors were mean plot mineral soil cover, mean rSOL depth, mean canopy combustion, stand age, and proportion of pre-fire black spruce. Seedling densities per m^2^ were rounded to whole numbers to meet requirements for a binomial model.

#### How do Seedling Densities Respond to Environmental Variation and Reduced Herbivory Under Relaxed Constraints of Seed Availability?

##### a. Natural Regeneration Plots

To assess drivers of seedling densities under natural conditions, we used natural seedling counts from the 219 plots and modeled black spruce and jack pine seedling densities for each ecozone. Aspen was only modeled on the Plains and birch was only modeled on the Shield due to their abundance limitations. For each species, models used seedling counts in each quadrat (that is, density of seedlings per m^2^) as the response. We used a mixture model approach to assess drivers of seedling densities, which includes a Bernoulli zero-inflation (ZI) component to model the probability of zero counts and a conditional component to model the expected count (Zuur and others 2009; Blasco-Moreno and others 2019). The conditional component assumed Poisson errors with logarithmic link. We selected variables for each component of the model based on consideration of their likely importance in each of the count and ZI components. For all species, predictors in both model components were percent mineral soil cover and rSOL depth. Conifer models also included measures of seed availability in both components: canopy combustion and pre-fire proportion of stems of black spruce or jack pine, which correlated with basal area (black spruce: *r* = 0.58; jack pine: *r* = 0.76). Stand age was assumed to be only important for the ZI-component but not the conditional component and attempts to include it in both caused non-convergence. A random effect for plot was included in both components.

##### b. Seed Addition Experimental Plots

We used the 30 experimental seed addition plots on the Plains to assess drivers of seedling establishment herbivory for black spruce, jack pine, aspen, and birch, in the absence of seed limitation and of vertebrate granivory. Models used the seedling counts in each sub-quadrat scaled to density of seedlings per m^2^ as the response. As above, we used a mixture model approach to assess drivers of seedling densities, with a ZI component (Bernoulli) and a conditional component (Poisson errors with logarithmic link). Predictors in both ZI and conditional components included a binary treatment factor (seeded or not), percent mineral soil cover, and rSOL depth. Conditional components additionally included a binary factor for exclosure (1 for exclosed, 0 otherwise). The seeded treatment was not included in the birch model because there were no naturally occurring birch seedlings in these plots. Models for conifer species included measures of seed availability in both components: canopy combustion and pre-fire proportion of stems of black spruce or jack pine, with stand age only in the ZI-component. We added an offset term log(subplot area) to the conditional component so the response scale had units of seedlings per m^2^. We included a random effect for plot in both components of all models.

## Results

### What are the Conditions that Promote Black Spruce Resilience?

Black spruce was dominant pre-fire at 86/133 plots in the Plains and 63/86 plots in the Shield. Post-fire, black spruce lost dominance at 57 (66%) and 26 (41%) plots in the Plains and Shield, respectively (Figure S5). In plots where black spruce was not dominant in the pre-fire stand, it gained dominance at only six (13%) and one (< 1%) plots in the plains and Shield, respectively. Jack pine, on the other hand, lost dominance at six (27%) and four (50%) plots, respectively. Where jack pine was not dominant before fire, it gained dominance at 32 (29%) plots in the Plains but only two plots in the shield (< 1%). In both ecozones, black spruce regularly lost dominance to broadleaf species, with aspen gaining dominance in the Plains at 21 (16%) plots and birch gaining dominance in the Shield at 30 (35%) plots.

**Figure 2:**
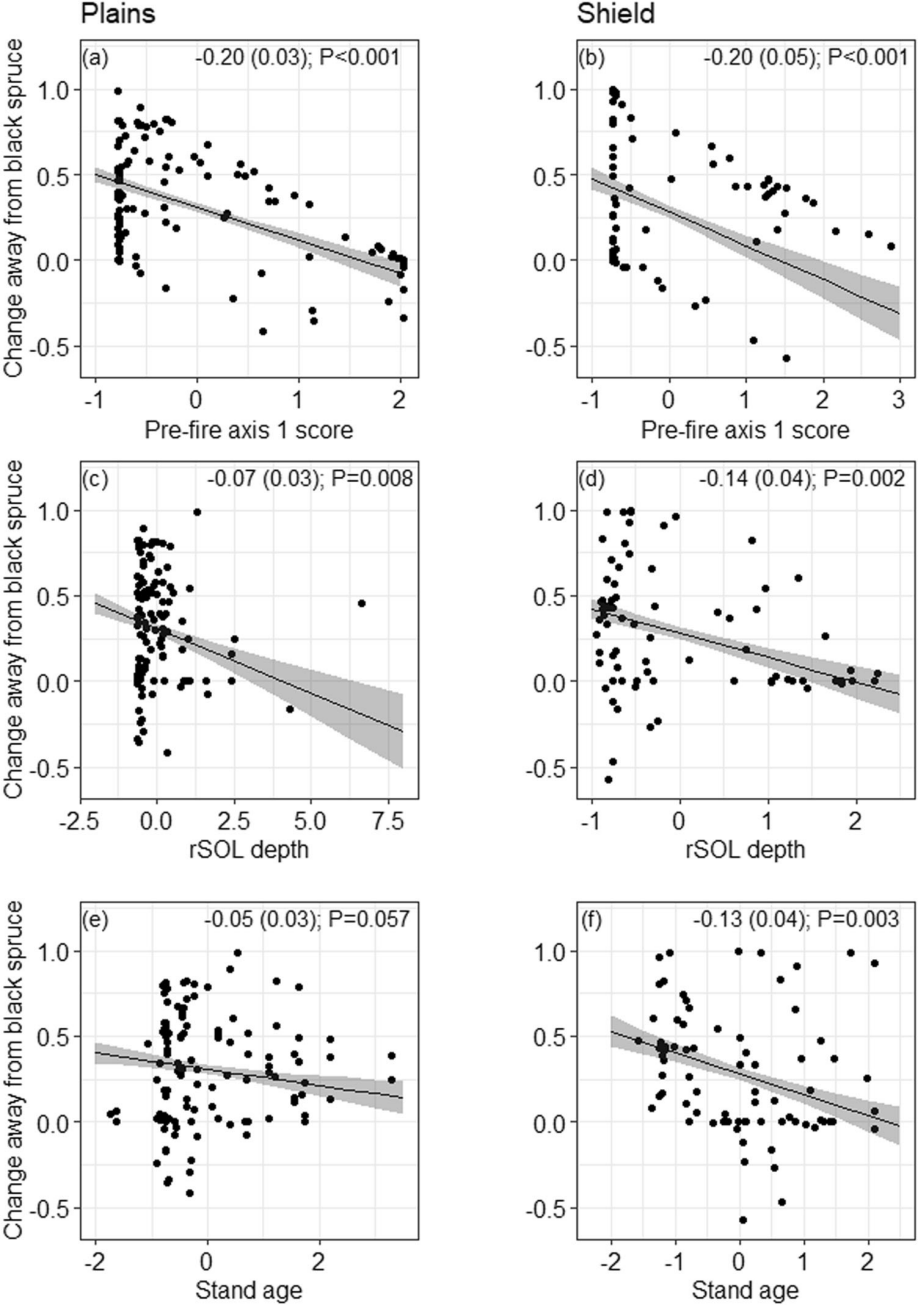
Covariates of forest compositional change under natural regeneration (no seed addition). Change away from black spruce on the *y*-axis show the change in pre-fire to post-fire composition along the first ordination axis in Figure 1, plotted against the original pre-fire composition (axis 1 score; low values represent pre-fire dominance of black spruce) on the Taiga Plains (**a**) and Taiga Shield (**b**); residual soil organic layer (rSOL) depths for the Plains (**c**) and Shield (**d**); and estimated stand age (time since last fire) for the Plains (**e**) and Shield (**f**). Regression lines are shown as solid lines with standard errors in gray, and coefficients with standard errors and *P*-values are shown at the top of each panel. Full model summaries are given in Table S3.

Relative changes in pre- to post-fire species composition were captured on a continuous scale by PCoA ordination of multivariate species proportions and reflect changes in composition but not necessarily shifts in dominance (Figures 1 and S4). Most plots had a compositional shift away from black spruce after fire: 82% (98/120) of plots in the Plains and 68% (54/79) plots in the Shield. Plots in the Plains shifted from black spruce toward jack pine or toward aspen, with post-fire seedling communities representing a full gradient of mixtures of spruce, pine, and aspen (Figure 1). In the Shield, shifts were predominantly from black spruce to birch and less commonly from jack pine to birch. Plots where black spruce was most abundant (lower axis scores in Figure 1) mathematically had the greatest potential for large shifts away from black spruce, resulting in a significant association between pre-fire composition and the magnitude of the shift away from black spruce (Figure 2). There were greater reductions in black spruce dominance after fire in plots with shallower rSOL (associated with high fire severity) and those burned at a younger age (Figure 2; Table S3).

Black spruce was more likely to dominate seedling counts where there was deeper rSOL and more pre-fire black spruce (Table S4). In the Shield, black spruce seedlings tended to dominate at sites with greater canopy combustion, however overall mean canopy combustion was lower on the Shield than on the Plains (Table S1).

### How do Seedling Densities Respond to Environmental Variation and Reduced Herbivory Under Relaxed Constraints of Seed Availability?

#### a. Natural Regeneration Plots

**Figure 3:**
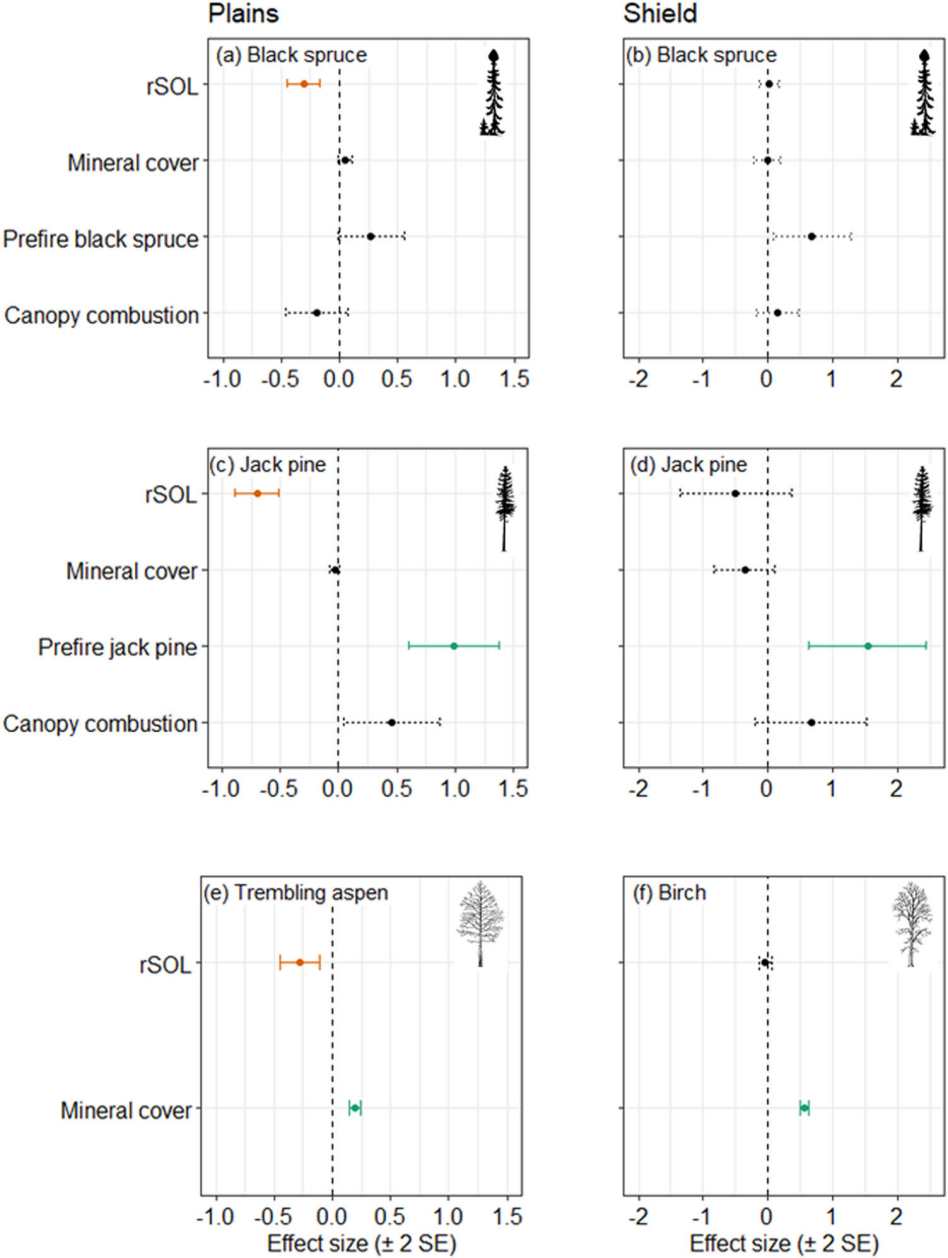
Under natural regeneration (no seed addition): Estimated effect sizes in the conditional count component of zero inflated mixed effects models of seedling counts in naturally regenerating burned boreal forest plots of the Taiga Plains (**a**, **c**, **e**) and Taiga Shield (**b**, **d**, **f**), Northwest Territories, Canada. Shown are standardized parameter estimates  ± 2 standard errors (SE). Statistically significant effects (*P*_adj_ < 0.05) are shown as solid lines, with green and orange indicating positive and negative relationships, respectively. rSOL: residual soil organic layer. Full model summaries are reported in Table S5.

Across the 219 naturally regenerating plots, predictors of seedling densities differed among species, and within species between ecozones (Figure 3, Table S5). There were significantly fewer seedlings associated with deeper rSOL for all species in the Plains. In the Shield however, rSOL was not related to conditional seedling counts, but the probability of zero seedlings of black spruce decreased with rSOL depth (Table S5). On the Plains, the probability of zero seedlings increased with stand age and decreased with the proportion of pre-fire black spruce. No variables predicted seedling presence of jack pine or broadleaf seedlings in either ecozone. There were more jack pine seedlings where there was more jack pine in both ecozones. There were more broadleaf seedlings (aspen on the Plains and birch on the Shield) where there was more exposed mineral soil (that is, seedbed; Figure 3).

#### b. Seed Addition Experimental Plots

**Figure 4:**
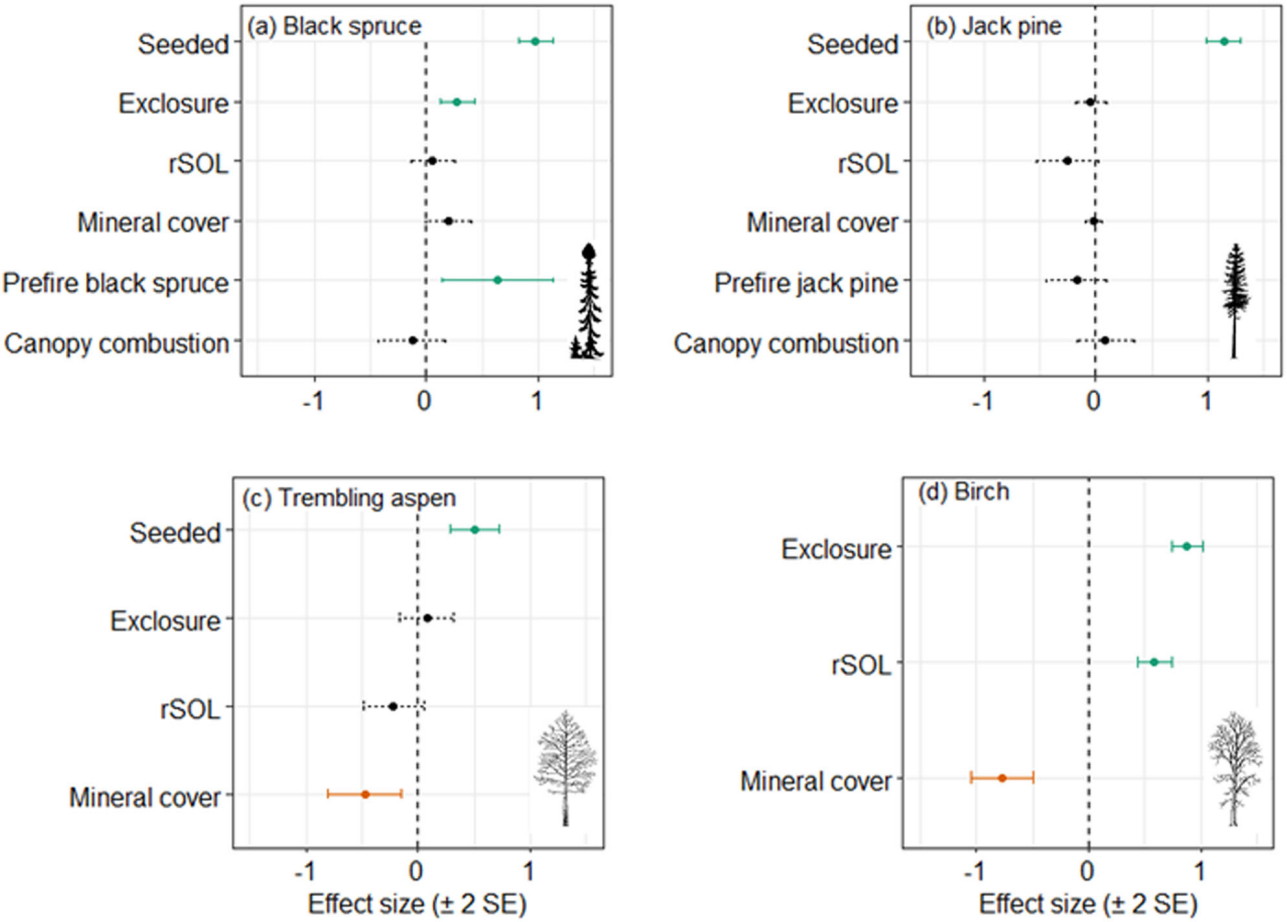
With seed addition: Effect sizes for the conditional count component of zero inflated mixed effects models of seedling counts at 30 plots where experimental seed addition occurred, within burned boreal forests of the Taiga Plains, Northwest Territories, Canada. Shown are standardised parameter estimates ± 2 standard errors (SE). Statistically significant effects (*P*_adj_ < 0.05) are shown as solid colored lines, with green and orange indicating positive and negative relationships, respectively. There is no effect size for the seeded treatment for birch because there were no naturally occurring birch seedlings. rSOL: residual soil organic layer. Full model summaries are reported in Table S6.

**Figure 5:**
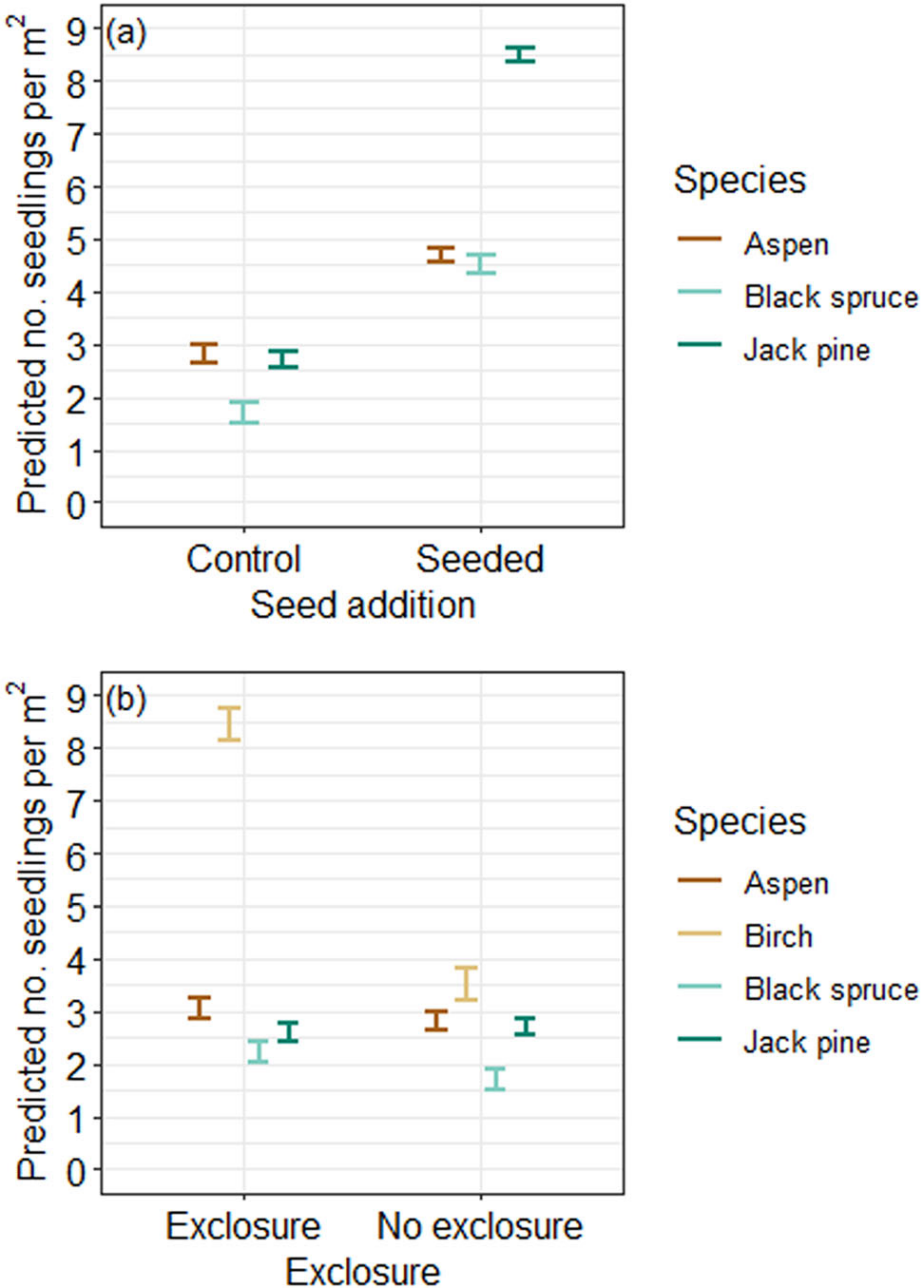
With seed addition: Predicted number of seedlings per m^2^ (± SE) from zero-inflated model for each species for seed addition treatment **a** and exclosure treatment **b** for 30 plots for seed addition experiment in burned boreal forests of the Taiga Plains, Northwest Territories, Canada. Note that there were no naturally occurring birch seedlings in our plots on the plains so the seed addition treatment was not included in this model. Full model outputs are in Table S6.

The positive effect of seed addition at the 30 experimental plots suggests that natural seedling establishment is seed-limited for all four species (Figure 4). Seed addition increased seedling density by a factor of 2.7 for black spruce, 3.1 for jack pine and 1.7 for aspen at the 30 experimental plots (Figure 5); the absence of natural birch recruitment means the effects of seed addition could not be estimated for that species. The seed addition treatment was significant in the zero-inflation component only for jack pine (Table S6), where seed addition increased the probability of presence.

Exclosures that provided barriers to vertebrates (mammals and birds) at the experimental plots significantly increased numbers of black spruce seedlings by a factor of 1.3 and birch by a factor of 2.1 (Figure 5). Black spruce seedlings had a lower probability of zero seedlings and more seedlings where there was more pre-fire black spruce. For jack pine, a greater probability of zero seedlings was associated with less pre-fire jack pine and deeper rSOL. For aspen and birch, greater mineral soil cover caused fewer seedlings. There were more birch seedlings in deeper rSOL (Figure 4; Table S6).

## Discussion

Our combination of observational and experimental data demonstrates that ecological memory is a key determinant of black spruce resilience after fire, primarily through material legacies that affect seed availability and seedbed quality, followed by information legacies that shape species-specific responses to environmental constraints. Black spruce recovery was reduced at sites with weaker material legacies in the form of shallower rSOL and less pre-fire black spruce available as a seed source. The seed addition experiment supported the importance of material legacies, specifically seed supply, as a primary mechanism underlying regeneration success for all species; adding seeds exceeded the magnitude of effects for all measured environmental and biotic factors on seedling densities. This clearly demonstrates that when legacy constraints on seed availability are relaxed, other species have strong potential to recruit into stands formerly dominated by black spruce and environmental constraints alone are insufficient to support black spruce dominance during the critical seedling regeneration phase after fire. Therefore, as other species become more common across the landscape and provide an increased source for seed dispersal, they may overwhelm black spruce resilience. An exception may be areas that can retain material legacies, such as thick rSOL, that preclude establishment or competitive dominance of species other than black spruce, or information legacies of adaptations to soil or climate conditions that prevent competing species from successful growth following recruitment (for example, Shenoy and others 2013). Our study demonstrating the strong role of legacy effects for black spruce resilience has two broad implications: (a) changes in composition in a changing environment will exhibit lagged effects depending on how well legacy effects are preserved, and (b) direct effects of fire on material legacies are an important mechanism eroding black spruce recovery following wildfire and resulting in alternate successional pathways.

Our results suggest that material legacies affecting seed availability and seedbed quality are a primary mechanism underlying black spruce recovery following fire. Natural seedling recruitment shows black spruce is most resilient where there was a high proportion of black spruce before fire to provide abundant seeds for self-replacement. The seed addition study provided direct evidence that seed availability was far more important than other abiotic or biotic factors likely to impact black spruce recruitment, similar to patterns observed in Alaska and Yukon (Brown and others 2015). We also showed how effects of fire characteristics on material legacies provide several mechanisms by which changes in fire regime due to climate change may directly alter forest resilience to fire. Here, we found that high canopy combustion reduced the proportion of seedlings that were black spruce, probably because of pre-dispersal seed mortality (Splawinski and others 2019). Secondly, short fire return intervals caused stands to burn at a young age and were associated with shifts away from black spruce dominance. There is accumulating evidence that short fire return intervals, which are increasingly common with climate warming and drying (Bowman and others 2020), disrupt the resilience of conifers that rely on local seed rain for recruitment (Brown and Johnstone 2012; Turner and others 2019; Whitman and others 2019) because there is not enough time to build a viable seedbank for self-replacement (Enright and others 2015; Nolan and others 2021). For non-serotinous species that rely on dispersal to colonize, fire patch size and distance to unburned seed sources become important (Foster and King 1986; Gill and others 2017). At our sites, aspen has the smallest seeds and therefore the greatest potential for long-distance dispersal, followed by birch, black spruce, then jack pine. Natural range shifts and human effects on species distributions, such as disturbance corridors, will also alter seed availability and are therefore expected to impact compositional shifts after fire.

Abiotic factors have direct and interactive effects on the legacies that affect black spruce resilience, highlighting the role of environmental heterogeneity in determining post-fire stand composition. In particular, we hypothesize that moisture availability is a key abiotic factor that interacts with ecological legacies to shape seedling recruitment after fire. Black spruce resilience is best supported in wet areas with deep rSOL because it can recruit and grow successfully under conditions of cold soils and low nutrient turnover that more strongly constrain growth of other species (Van Cleve and others 1983; Johnstone and others 2010b). We found that most plots exhibiting resilience of black spruce after fire were in wet areas with thick soil organic layers, which tend to experience low fire severity and retain proportionally more SOL (Walker and others 2018b). While mineral soil can often signal good quality seedbeds, plots in our seed addition study with high mineral cover corresponded to xeric sites on sandy soils, which likely limited recruitment where seeds were added (Figures 4 and S6). Dry site conditions were probably accentuated by the unusually high temperatures in the weeks following experimental seeding in June 2016 (Figure S7; Wang and others 2012). The combination of drying of the landscape and fire activity is therefore expected to lead to more widespread shifts in vegetation. On paleoecological timescales, warming and associated increases in fire activity 2000–7000 years ago led to replacement of spruce with jack pine or broadleaf trees in eastern Canada (Remy and others 2017; Jensen and others 2021). During these widespread changing conditions in the past, spruce was most abundant following fire in areas with moist soils (Jensen and others 2021). Thus, climate warming may cause gradual transitions in vegetation but fire pushes sites to alternative stable states by rapidly eroding material legacies.

Our study demonstrates the combination of ecological memory and environmental heterogeneity in supporting the differentiation of the realized niche of tree species across the western North American boreal landscape. The study species overlap in their environmental tolerances and have relatively wide potential niches, but ecological memory in the form of material legacies appears to be a key factor in keeping species confined along environmental gradients. For example, in the absence of seed limitation, we demonstrated that broadleaf taxa can establish in high densities in sites with deep rSOL as long as there is enough moisture in the soil. Localized patches of birch can be maintained at high densities in waterlogged areas for many years and are thought to exclude establishment of black spruce even where there are abundant seed sources (Dearborn and others 2021), suggesting black spruce may not necessarily outcompete birch in wet areas. The success of birch in the seed addition experiment strongly suggests this species is not rare in the Plains due to environmental characteristics but due to lack of propagules. Thus, climate and fire-induced changes in species distributions that accumulate over time could further erode black spruce resilience in the long term.

Our experiment demonstrated that biotic effects, in the form of granivory or herbivory by vertebrates, can significantly impact material legacies to reduce seedling densities of black spruce and birch. Such effects are likely to be contingent on local vertebrate populations and may be biologically important for recruitment under low seed availability or marginal environmental conditions (for example, Urli and others 2016; Olnes and others 2017). Despite jack pine seeds being more palatable than those of black spruce (Martell 1979) and our frequent observations of herbivory on jack pine seedlings (likely by lagomorphs), jack pine seedling densities were unimpacted by exclosures. Jack pine’s fast growth may enhance resistance to herbivory, providing an additional advantage over black spruce for post-fire expansion where it is present. Studies assessing the impacts of granivory and herbivory on seed availability and recruitment in the boreal forest are equivocal. Post-dispersal granivory reduced seed numbers by up to 58% in Quebec (Côté and others 2003), but vertebrate herbivory had no detectable impact on post-fire tree densities or biomass in Alaska 13 years after fire (Johnstone and others 2020). In Labrador, desiccation caused more deaths of transplanted seedlings than herbivore exclusion (Moss and Hermanutz 2009). Our study was not designed to assess impacts of invertebrates, which may also affect seedling densities (Hargreaves and others 2019). Fluctuating populations of seed or seedling consumers will add stochasticity to observed recruitment patterns (Zwolak and others 2012; Olnes and Kielland 2017). Overall, more experimental studies are needed to understand the role of biotic interactions on seedling recruitment and post-fire plant composition, including differential responses of tree species to mutualists or pathogens (for example, Day and others 2020). At the very least, our study provides evidence of granivory/herbivory as a species-specific biological hurdle that requires additional seed inputs to overcome for successful seedling recruitment.

We constrained our focus to testing mechanisms of black spruce resilience because this species has been self-replacing in boreal forests across North America under historical fire conditions for much of the Holocene (MacDonald 2000; Girardin and others 2013; Kelly and others 2013). Fire return intervals in western boreal forests are typically too short for successional replacement from broadleaf to conifer dominance to be observed (Bergeron and Dubue 1988; Fastie and others 2002; Kurkowski and others 2008). Where seed is available after fire, loss of black spruce resilience is likely to translate into alternative patterns of forest dominance, rather than a state change to non-forest. For example, jack pine has information legacies that make it apparently better adapted to shorter fire return intervals and severe fires (Burns and Honkala 1990; Lavoie and Sirois 1998; Greene and others 2004). Shifts from spruce to alternative dominance by pine or broadleaf species are likely to be maintained if new material legacies are created that support recovery of those types after fire (Johnstone and others 2010b). However, in extreme conditions conifer stands may transition to non-forested states when recruitment failure occurs (Brown and Johnstone 2012), particularly in association with drought and short disturbance return intervals (Whitman and others 2019; Baltzer and others 2021).

When considering ecosystem-level implications of changes in black spruce resilience, shifts away from black spruce dominance to another conifer may have fewer functional implications than shifts toward broadleaf taxa. Compared to black spruce, jack pine stands have similar landscape flammability (Cumming 2001) and have similar biomass of caribou lichen forage (Boan and others 2013; Greuel and others 2021). However, a shift from black spruce to either broadleaf or jack pine will likely reduce belowground carbon storage because of greater proportional allocation of carbon aboveground (Alexander and Mack 2016; Walker and others 2018b; Mack and others 2021), and broadleaf-dominated forests have sparser lichens than black spruce stands (Boan and others 2013; Greuel and others 2021). Finally, large-scale shifts from conifer to broadleaf tree cover in boreal forests may cause negative feedbacks to climate warming through increased albedo (Beck and others 2011; Wit and others 2014) and reduce fire spread on the landscape by lowering flammability (Krawchuk and Cumming 2011; Girardin and others 2013; Marchal and others 2020), although climatic warming and drying may overcome this effect. Given the high frequency of state changes away from black spruce (Baltzer and others 2021), an improved understanding of the functional implications of these shifts is needed.

The mechanisms here are generalizable to post-fire plant communities in many biomes: recovery after fire relies on a combination of ecological memory, principally in the form of material legacies, and environmental conditions. Warming and drying under current climate change will reduce soil moisture availability for regeneration of forests in many areas of the globe (Hansen and others 2018; Stevens-Rumann and others 2018). Increased fire activity beyond historical norms due to climate change will further erode ecological memory transmitted through material legacies (Bowman and others 2016; Turner and others 2019; Whitman and others 2019; Nolan and others 2021). Species that are not tolerant or competitive across wide environmental ranges will be most negatively impacted by changing climate. Thus, species adaptations and traits form an important information legacy. In the boreal forest context, small-seeded broadleaf species have previously been excluded from black spruce sites due to strong effects of material legacies in the form of thick rSOL, which constrain both recruitment and subsequent growth of broadleaf species. Jack pine may have been excluded due to lack of propagule legacies. As material legacies change, other information legacies may become important.

Overall, our study shows that resilience of black spruce to fire has been supported by ecological legacies combined with environmental constraints on the establishment success of other boreal tree species. Black spruce resilience is vulnerable to effects of increased fire activity, which erode material legacies and reduce the potential for self-replacement; high fire severity increases combustion of seedbanks and short fire return intervals lead to inadequate time to develop these aerial seedbanks. Moreover, black spruce relies on being able to grow in areas with deep soil organic layers because other species are less competitive in these conditions. However, drying landscapes under climate change will likely expand conditions favorable for establishment of other species. In the short term, black spruce competitor species will increase following fire by reducing environmental constraints, and over longer timeframes their establishment will be reinforced by having more seed available; both mechanisms facilitate vegetation transitions away from black spruce. Our results demonstrate the potential importance of biotic interactions in creating species-specific barriers to establishment. Transitions away from black spruce dominance have functional implications for the boreal biome such as changes in flammability, albedo, and distribution and storage of carbon above and belowground. Functional impacts differ depending on which species become dominant in the place of black spruce but given the current dominance of this species in boreal North America, characterization and upscaling these functional changes is urgently needed.

## Supplementary Information

Below is the link to the electronic supplementary material.

Supplementary file1 (DOCX 1135 kb)

Supplementary file2 (r 36 kb)

Supplementary file3 (DOCX 12 kb)

## Data Availability

Some data are already published in the following archives: Baltzer, J. L., Day, N. J., White, A. L., Reid, K. A., Degré-Timmons, G., Johnstone, J. F., Cumming, S. G., Mack, M. C., Turetsky, M. R., & Walker, X. J. (2020). *Vascular Plant Community Data for Northwest Territories, Canada*. Dryad 10.5061/dryad.76hdr7sth. Walker, X.J., B.M. Rogers, J.L. Baltzer, S.R. Cumming, N.J. Day, S.J. Goetz, J.F. Johnstone, M.R. Turetsky, and M.C. Mack. 2018. ABoVE: Wildfire Carbon Emissions and Burned Plot Characteristics, NWT, CA, 2014–2016. ORNL DAAC, Oak Ridge, Tennessee, USA. 10.3334/ORNLDAAC/1561. - remaining data will be added to this dryad record upon manuscript acceptance.
