# Supplementary material for: Material Legacies and Environmental Constraints Underlie Fire Resilience of a Dominant Boreal Forest Type
Source: Ecosystems. 2022 Jun 29;26(3):473–90. doi: 10.1007/s10021-022-00772-7 (PMC10167110; doi:10.1007/s10021-022-00772-7)
Supplement: Supplementary file 1 — Supplementary file1 (DOCX 1135 kb) [file 10021_2022_772_MOESM1_ESM.docx]

# **Supplementary figures and tables for:**

Material legacies and environmental constraints underlie post-fire resilience of a dominant boreal forest type

Nicola J Day, Kirsten A Reid, Jill F Johnstone, Steven G Cumming, Michelle C Mack, Merritt R Turetsky, Xanthe J Walker, Jennifer L Baltzer

## **Figures**


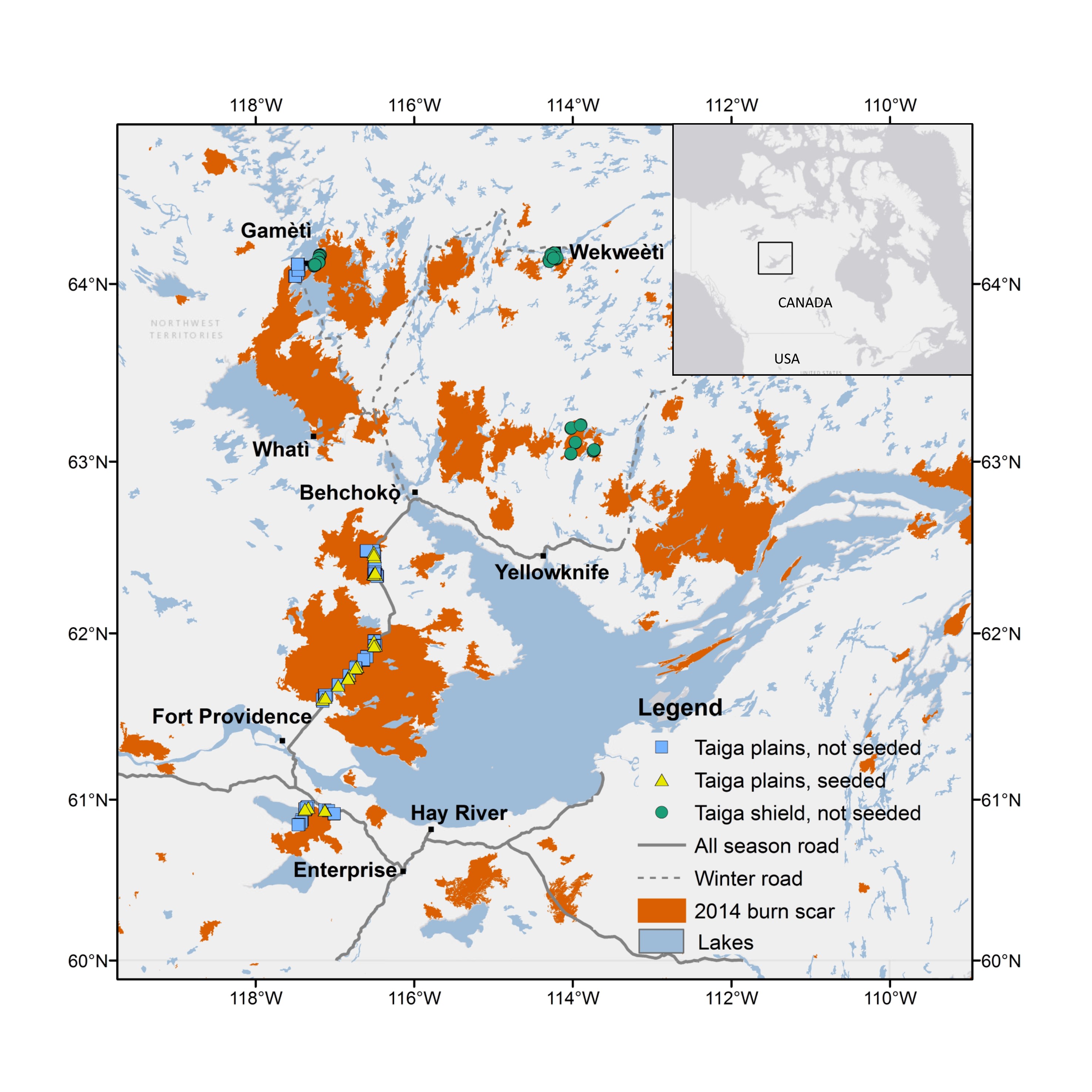


Fig. S1. Map of sampling locations of boreal forests within the Northwest Territories, Canada, spanning two ecozones. Plots with natural regeneration (n=219) are shown as blue squares and green circles; plots with seed addition (n=30) shown as yellow triangles.


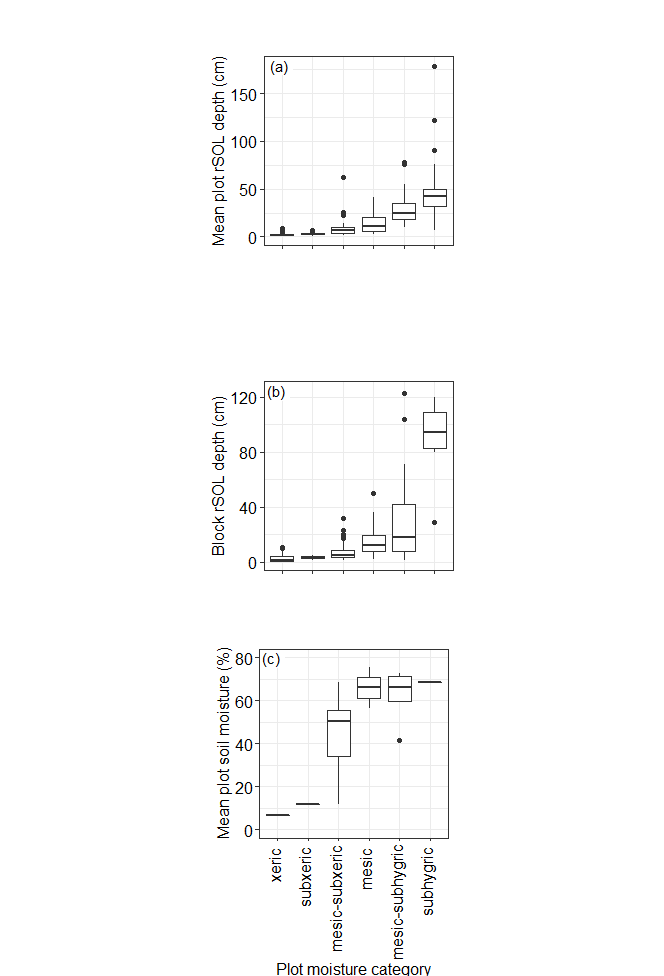


Fig. S2. Positive relationships between soil moisture category and mean plot residual soil organic layer (rSOL) depth at the plot level for 219 plots (a), rSOL depth within blocks at the for 30 plots where seeds were experimentally added (b), and mean plot gravimetric soil moisture for 17 plots where seeds were experimentally added (c) in burned boreal forests in the Northwest Territories, Canada. Gravimetric soil moisture data is from soils collected from the top 5 cm for 17 plots in summer 2015.


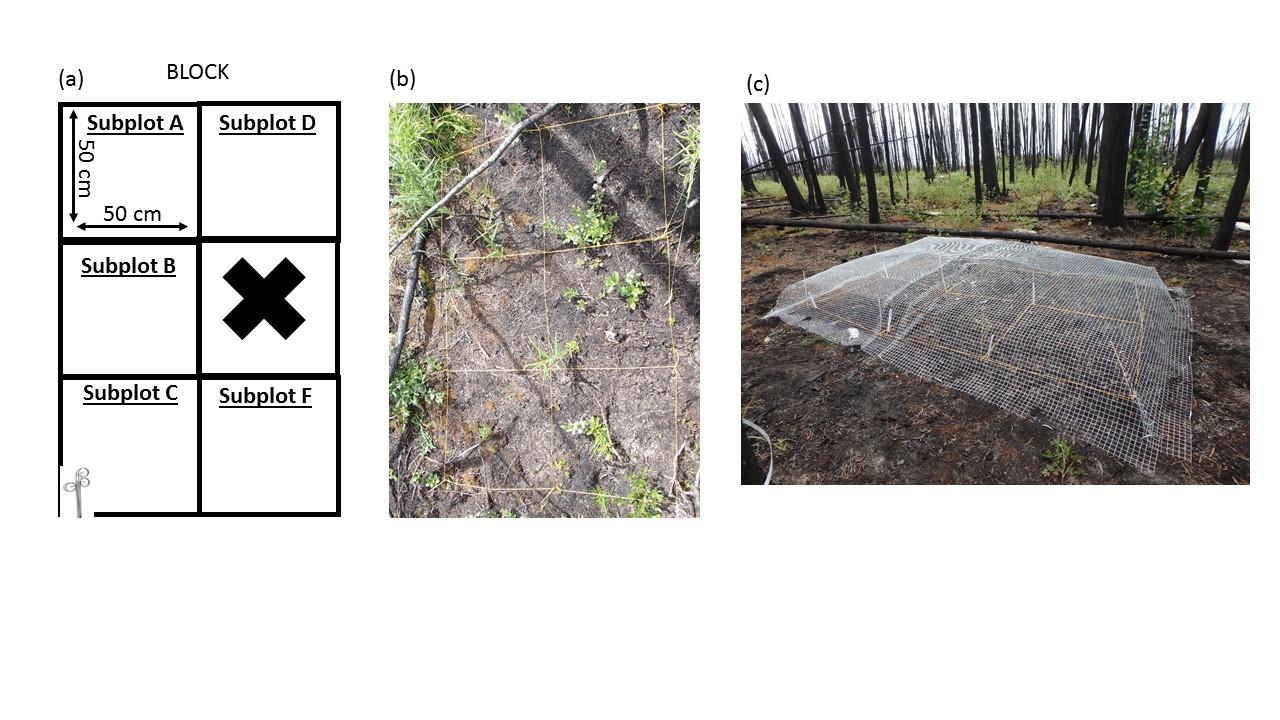


Fig. S3. Set up of seeding experiment blocks at 30 locations in burned boreal forests in the Northwest Territories, Canada, showing the block schematic (a), block in the field (b), and mammal exclosure (c). The subplot marked “X” is where residual soil organic layer (rSOL) depth was measured destructively.


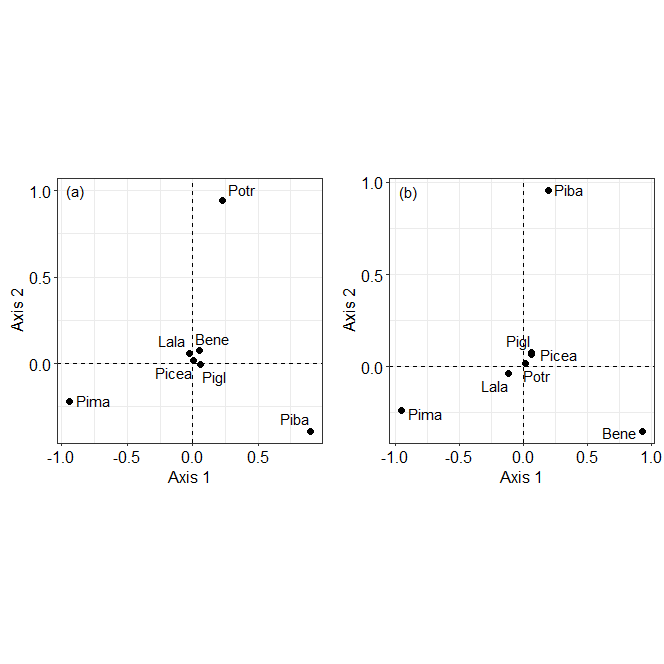
 Fig. S4. Under natural regeneration (no seed addition): Species scores for principal co-ordinates analysis ordination on pairwise Bray-Curtis distance for plots on the Plains (a) and Shield (b) in burned boreal forests of the Northwest Territories, Canada. Pima: *Picea mariana* (black spruce); Piba: *Pinus banksiana* (jack pine); Potr: *Populus tremuloides* (aspen); Bene: *Betula papyrifera* (paper birch); Lala: *Larix laricina* (larch); Pigl: *Picea glauca* (white spruce); Picea: *Picea* spp. (black or white spruce, due to difficulties determining the difference in young seedlings and some small burned trees). See Fig. 1 for graphs with site scores.


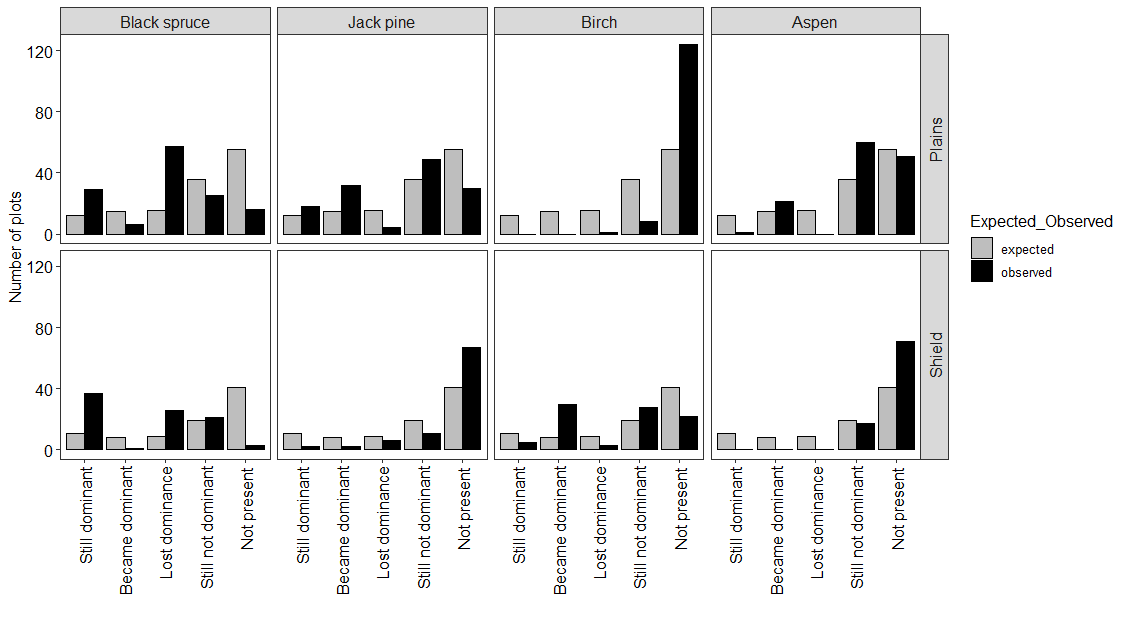


Fig. S5. Under natural regeneration (no seed addition): Change in dominance categories post-fire (seedlings) compared to pre-fire (trees) for each species on the Plains (n=133) and the Shield (n=86) in burned boreal forests of the Northwest Territories, Canada. A species was considered dominant at a given plot if it made up 50% of the stems. Became dominant: species was not dominant before fire but became dominant post-fire; Lost dominance: species was dominant pre-fire and was not dominant post-fire; Still dominant: species was dominant both pre- and post-fire; Still not dominant: species was not dominant pre- and post-fire. Chi-square tests tested whether the observed distribution of each species in each category differs significantly from what would be expected in the population as a whole. Results showed that there were significant differences between observed number of plots in each category of dominance change compared to the expected number of plots (Plains: χ^2^=412.39, *P*<0.001; Shield: χ^2^=298.09, *P*<0.001). In both ecozones, black spruce lost dominance at more plots than expected from the χ^2^ contingency table test. On the Plains, jack pine became dominant at more plots than expected (based on contingency tables and Chi-square tests) and to a lesser degree aspen also became dominant at more plots than expected. On the Shield, birch became dominant at more plots than expected.


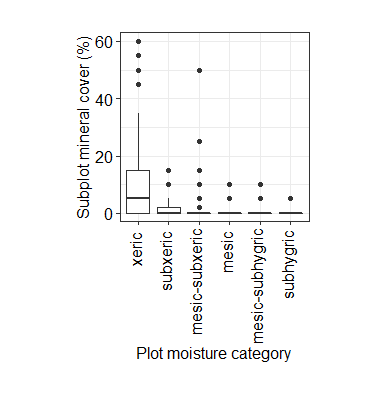


Fig. S6. Subplot mineral soil cover (%) against plot moisture category for 30 plots for seed addition experiment in burned boreal forests of the Taiga Plains, Northwest Territories, Canada.


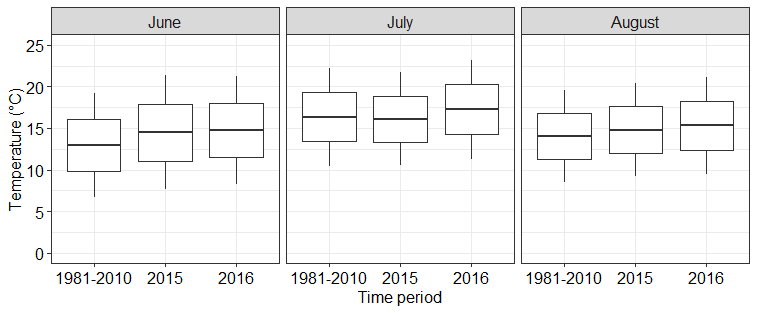


Fig. S7. Boxplots of temperature for growing season months (June, July, August) showing 30-year normals (1981-2010) and 2015 and 2016 values for 30 plots in burned boreal forests of the Taiga Plains, Northwest Territories, Canada. The 2015 and 2016 values correspond to important recruitment periods following the 2014 fires and the summer (2016) during which the seeding experiment was conducted. The box represents the interquartile range, the band represents the median, and the whiskers indicate data within 1.5 times the interquartile range. Data were derived for each plot location from ClimateWNA (Wang and others 2012).

## **Tables**

Table S1. Plot-level mean, minimum, and maximum values for predictor variables used in statistical analyses for all 219 plots in boreal forests in the Northwest Territories, Canada, 86 plots on the Taiga Shield (Shield), 133 plots on the Taiga Plains (Plains), and the 30 plots on the Plains where the seed addition experiment was undertaken. Fire severity is proportion soil organic layer combusted. rSOL=residual soil organic layer.

| **Variable** | **All (n=219)** | **Shield (n=86)** | **Plains (n=133)** | **Seed addition**  **(Plains; n=30)** |
| --- | --- | --- | --- | --- |
| Elevation (m) | 264 (189, 408) | 304 (189, 408) | 237 (191, 292) | 238 (192, 278) |
| Stand age (time since previous fire; years) | 102 (19, 232) | 100 (19, 205) | 103 (30, 232) | 107 (70, 232) |
| Proportion pre-fire black spruce | 0.63 (0,1) | 0.68 (0, 1) | 0.59 (0, 1) | 0.68 (0, 1) |
| Proportion pre-fire jack pine | 0.14 (0, 1) | 0.08 (0, 1) | 0.19 (0, 1) | 0.18 (0, 1) |
| Proportion other | 0.23 (0, 1) | 0.24 (0, 1) | 0.22 (0, 1) | 0.14 (0, 1) |
| rSOL depth (cm) | 15.84 (0.10, 178.25) | 13.97 (0.25, 50.20) | 17.05 (0.10, 178.25) | 19.21 (0.35, 77.85) |
| Proportion SOL combusted | 0.53 (0.9, 1) | 0.54 (0.15, 0.96) | 0.53 (0.09, 1) | 0.54 (0.21, 1) |
| Pre-fire SOL depth (cm) | 22.04 (5.78, 72.28) | 22.93 (6.34, 59.15) | 21.46 (5.78, 72.28) | 21.70 (5.78, 56.10) |
| Mineral soil cover (%) | 6.32 (0, 100) | 6.31 (0, 100) | 6.33 (0, 100) | 2.16 (0, 60) |
| Canopy combustion | 1.89 (0, 3) | 1.49 (0.38, 2.89) | 2.09 (0, 3) | 2.40 (1.14, 3) |

Table S2. Characteristics of the experimental seed addition study undertaken at 30 plots in burned boreal forests of the Taiga Plains (Plains), Northwest Territories, Canada. SE: standard error of the mean.

| **Species** | **Provenance** | **Mass of seeds sown (g/subplot)** | **Viable seeds sown (no. seeds/subplot)** | **Viability** | **Mean seedling: viable seed ratio (SE)** |
| --- | --- | --- | --- | --- | --- |
| Black spruce | Plains (multiple locations between N61.61°, W-117.15° and N62.49°, W-116.60°);  Shield (N62.55°, W-113.91°), Northwest Territories | 0.12 | 100 | 72% | 0.047 (0.007) |
| Jack pine | Plains (multiple locations between N61.61°, W-117.15° and N62.49°, W-116.60°);  Shield (N62.55°, W-113.91°), Northwest Territories | 0.42 | 100 | 69% | 0.087 (0.009) |
| Aspen | N61.35°, W-117.66°, Northwest Territories;  N55.10°, W-105.3°, Saskatchewan | 0.17 | 1500 | 90% | 0.002 (0.0005) |
| Birch | Hay River, Plains, Northwest Territories (N60.83°, W-115.78°) | 0.13 | 300 | 45% | 0.024 (0.004) |

Estimation of seed viability: Six replicates of 50 seeds each were cold-wet stratified on paper towels at 4°C (jack pine, black spruce: 21 days; birch: 60 days; aspen: no stratification). Seeds were germinated in 50:50 sterile soil:sand mix for 4 wks in a growth chamber with 20/4 h of day/night at 21/18°C.

Table S3. Under natural regeneration (no seed addition): Results from linear models assessing drivers of direction and distances along the main axis of change away from black spruce (first PCoA axis) of plot seedling composition from pre-fire composition for burned boreal forests in the Northwest Territories, Canada (n=219). Standardised values for the predictors are shown. rSOL: residual soil organic layer. SE: standard error of the mean. Bold denotes significant relationship at *P*<0.05. Predicted values for significant variables are shown in Fig. 2.

| **Ecozone** | **Predictor** | **Estimate** | **SE** | ***t*** | ***P*** |
| --- | --- | --- | --- | --- | --- |
| Plains | (Intercept) | 0.31 | 0.03 | 12.27 | <0.001 |
|  | Mineral soil | 0.01 | 0.03 | 0.35 | 0.724 |
|  | **rSOL** | **-0.08** | **0.03** | **-2.79** | **0.006** |
|  | Canopy combustion | 0.01 | 0.03 | 0.39 | 0.701 |
|  | Stand age | -0.05 | 0.03 | -1.77 | 0.080 |
|  | **Pre-fire axis 1** | **-0.19** | **0.03** | **-5.60** | **<0.001** |
|  | Pre-fire axis 2 | -0.02 | 0.03 | -0.90 | 0.371 |
| Shield | (Intercept) | 0.28 | 0.04 | 7.80 | <0.001 |
|  | Mineral soil | 0.05 | 0.04 | 1.13 | 0.263 |
|  | **rSOL** | **-0.14** | **0.04** | **-3.25** | **0.002** |
|  | Canopy combustion | -0.05 | 0.04 | -1.41 | 0.162 |
|  | **Stand age** | **-0.12** | **0.04** | **-2.92** | **0.005** |
|  | **Pre-fire axis 1** | **-0.20** | **0.05** | **-3.90** | **<0.001** |
|  | Pre-fire axis 2 | 0.03 | 0.04 | 0.83 | 0.409 |

Table S4. Under natural regeneration (no seed addition) and only plots where black spruce was present: Results from generalised linear models assessing drivers of the proportion of seedlings in a plot that were black spruce (binomial response) in burned boreal forests in the Northwest Territories, Canada. Standardised values for the predictors are shown, based on measurements of mineral soil proportion, rSOL (residual soil organic layer) in cm, canopy combustion class, stand age in years, and pre-fire black spruce as a proportion of total stems. SE: standard error of the mean. Bold denotes significant relationship at *P*<0.05. Sample sizes account for exclusion of plots with zero seedlings from the analyses.

| **Ecozone** | **Predictor** | **Estimate** | **SE** | ***Z*** | ***P*** |
| --- | --- | --- | --- | --- | --- |
| Plains (n=91) | (Intercept) | -1.31 | 0.05 | -24.84 | <0.001 |
|  | Mineral soil | -0.03 | 0.06 | -0.45 | 0.654 |
|  | **rSOL** | **0.28** | **0.06** | **4.46** | **<0.001** |
|  | Canopy combustion | 0.04 | 0.06 | 0.64 | 0.522 |
|  | Stand age | 0.06 | 0.06 | 1.09 | 0.278 |
|  | **Pre-fire black spruce** | **0.30** | **0.06** | **4.86** | **<0.001** |
| Shield (n=77) | (Intercept) | -1.44 | 0.12 | -11.87 | <0.001 |
|  | Mineral soil | -0.28 | 0.20 | -1.41 | 0.160 |
|  | **rSOL** | **0.51** | **0.14** | **3.65** | **<0.001** |
|  | **Canopy combustion** | **0.28** | **0.12** | **2.32** | **0.020** |
|  | Stand age | 0.13 | 0.11 | 1.16 | 0.247 |
|  | **Pre-fire black spruce** | **0.65** | **0.18** | **3.63** | **<0.001** |

Table S5. Under natural regeneration (no seed addition): Results from zero inflated mixed effect models for seedling counts for natural regeneration (no seed addition) across burned boreal forests of the Plains, Northwest Territories, Canada. Models were run at the quadrat level for 665 quadrats (in 133 plots) on the Taiga Plains (Plains) and 430 quadrats (in 86 plots) on the Taiga Shield (Shield). There are different numbers of quadrats for jack pine and aspen because outliers with high leverage were removed. Note that the zero-inflation component provides the probability of a zero (logit). rSOL: residual soil organic layer. SE: standard error of the mean. Bold denotes significant relationship at *P*_adj_<0.05. NA indicates variables that were not put in both components of each model. Random effects: Plains Black spruce conditional 0.89, ZI: 5.88; Shield black spruce conditional: 1.02, ZI: 0.72; Plains jack pine conditional: 3.65; Shield jack pine conditional: 5.61, ZI: 0.71; aspen conditional: 8.83, ZI: 0.18; birch conditional: 6.67.

|  |  |  | **Conditional** | | | | | **Zero-inflated** | | | | |
| --- | --- | --- | --- | --- | --- | --- | --- | --- | --- | --- | --- | --- |
| **Ecozone** | **Species** | **Predictor** | **Estimate** | **SE** | ***Z*** | **Adjusted *P*** | **Estimate** | | **SE** | ***Z*** | **Adjusted *P*** |  |
| Plains | Black spruce  (n=665) | **(Intercept)** | **1.50** | **0.12** | **12.13** | **<0.001** | -0.06 | | 0.25 | -0.24 | 0.840 |  |
|  |  | Mineral cover | 0.05 | 0.03 | 1.46 | 0.211 | 0.00 | | 0.20 | 0.02 | 0.986 |  |
|  |  | **rSOL depth** | **-0.31** | **0.07** | **-4.37** | **<0.001** | 0.39 | | 0.27 | 1.44 | 0.211 |  |
|  |  | **Pre-fire black spruce** | 0.27 | 0.14 | 1.88 | 0.129 | **-2.07** | | **0.43** | **-4.79** | **<0.001** |  |
|  |  | Canopy combustion | -0.20 | 0.13 | -1.46 | 0.211 | -0.25 | | 0.33 | -0.76 | 0.504 |  |
|  |  | **Stand age** |  | NA | NA | NA | **0.88** | | **0.29** | **3.02** | **0.009** |  |
|  | Jack pine | (Intercept) | 0.34 | 0.19 | 1.74 | 0.149 | -2.40 | | 0.35 | -6.94 | <0.001 |  |
|  | (n=663) | Mineral cover | -0.04 | 0.02 | -1.72 | 0.149 | -1.21 | | 0.86 | -1.41 | 0.212 |  |
|  |  | **rSOL depth** | **-0.70** | **0.10** | **-7.33** | **<0.001** | 0.61 | | 0.33 | 1.85 | 0.130 |  |
|  |  | **Pre-fire jack pine** | **0.99** | **0.20** | **5.05** | **<0.001** | -0.20 | | 0.31 | -0.64 | 0.564 |  |
|  |  | Canopy combustion | 0.46 | 0.21 | 2.20 | 0.078 | -0.39 | | 0.30 | -1.27 | 0.247 |  |
|  |  | Stand age | NA | NA | NA | NA | 0.49 | | 0.25 | 1.97 | 0.125 |  |
|  | Aspen | (Intercept) | -0.56 | 0.36 | -1.58 | 0.189 | -0.23 | | 0.17 | -1.37 | 0.216 |  |
|  | (n=663) | **Mineral cover** | **0.19** | **0.03** | **7.59** | **<0.001** | -0.29 | | 0.26 | -1.13 | 0.301 |  |
|  |  | **rSOL depth** | **-0.28** | **0.09** | **-3.22** | **0.004** | 0.37 | | 0.19 | 1.93 | 0.126 |  |
| Shield | Black spruce  (n=430) | (Intercept) | 0.38 | 0.22 | 1.74 | 0.287 | -0.18 | | 0.52 | -0.34 | 0.859 |  |
|  |  | Mineral cover | -0.01 | 0.10 | -0.06 | 0.955 | -0.95 | | 1.10 | -0.86 | 0.619 |  |
|  |  | **rSOL depth** | 0.01 | 0.08 | 0.15 | 0.954 | **-0.55** | | **0.20** | **-2.69** | **0.049** |  |
|  |  | Pre-fire black spruce | 0.68 | 0.30 | 2.26 | 0.134 | -0.06 | | 0.42 | -0.14 | 0.954 |  |
|  |  | Canopy combustion | 0.16 | 0.16 | 0.96 | 0.588 | 0.19 | | 0.23 | 0.85 | 0.619 |  |
|  |  | Stand age | NA | NA | NA | NA | -0.10 | | 0.21 | -0.50 | 0.775 |  |
|  | Jack pine | **(Intercept)** | **-3.80** | **1.09** | **-3.51** | **<0.001** | -1.42 | | 1.09 | -1.30 | 0.429 |  |
|  | (n=430) | Mineral cover | -0.36 | 0.24 | -1.53 | 0.321 | -5.17 | | 2.73 | -1.90 | 0.232 |  |
|  |  | rSOL depth | -0.50 | 0.43 | -1.15 | 0.496 | 0.05 | | 0.73 | 0.08 | 0.955 |  |
|  |  | **Pre-fire jack pine** | **1.54** | **0.45** | **3.39** | **0.009** | -0.31 | | 0.52 | -0.59 | 0.737 |  |
|  |  | Canopy combustion | 0.67 | 0.43 | 1.54 | 0.321 | 0.35 | | 0.51 | 0.69 | 0.689 |  |
|  |  | Stand age | NA | NA | NA | NA | -0.30 | | 0.63 | -0.47 | 0.775 |  |
|  | Birch | (Intercept) | -0.48 | 0.37 | -1.29 | 0.429 | -0.27 | | 0.18 | -1.53 | 0.321 |  |
|  | (n=430) | **Mineral cover** | **0.56** | **0.03** | **16.92** | **<0.001** | -0.15 | | 0.15 | -1.06 | 0.543 |  |
|  |  | rSOL depth | -0.04 | 0.05 | -0.72 | 0.689 | 0.44 | | 0.21 | 2.12 | 0.159 |  |

Table S6. Where seed addition occurred: Results from zero inflated mixed effect models for seedling counts where seed addition occurred in burned boreal forests of the Taiga Plains, Northwest Territories, Canada. The seeded treatment was not included for birch because there were no naturally occurring birch seedlings at the plots. There are different numbers of subplots for each species because jack pine was only added at 26 plots and blocks with outliers with high leverage were removed from other models. The zero-inflation component models the probability of a zero count, using a logit link. rSOL: residual soil organic layer. SE: standard error of the mean. Bold denotes significant relationship at *P*_adj_<0.05. NA indicates variables that were not put in both components of each model. Random effects: Black spruce conditional: 0.49, ZI: 0.23; Jack pine conditional: 0.38, ZI: 0.07; Aspen conditional: 0.34, ZI: 0.97; Birch conditional: 2.31.

|  |  | **Conditional model** | | | | **Zero-inflation model** | | | |
| --- | --- | --- | --- | --- | --- | --- | --- | --- | --- |
| **Species** | **Predictor** | **Estimate** | **SE** | ***Z*** | **Adjusted *P*** | **Estimate** | **SE** | ***Z*** | **Adjusted *P*** |
| Black spruce  N=336 | **(Intercept)** | **1.92** | **0.18** | **10.58** | **<0.001** | -0.17 | 0.26 | -0.67 | 0.668 |
|  | **Seed addition** | **0.98** | **0.08** | **12.52** | **<0.001** | -0.22 | 0.30 | -0.74 | 0.668 |
|  | Mineral soil | 0.20 | 0.10 | 1.89 | 0.128 | 0.07 | 0.20 | 0.35 | 0.796 |
|  | rSOL | 0.06 | 0.10 | 0.57 | 0.692 | -0.09 | 0.21 | -0.44 | 0.761 |
|  | **Pre-fire black spruce** | **0.64** | **0.25** | **2.59** | **0.024** | **-1.03** | **0.26** | **-3.88** | **<0.001** |
|  | Canopy combustion | -0.13 | 0.15 | -0.88 | 0.623 | -0.07 | 0.18 | -0.41 | 0.773 |
|  | **Exclosure** | **0.28** | **0.08** | **3.64** | **<0.001** | NA | NA | NA | NA |
|  | Stand age | NA | NA | NA | NA | 0.03 | 0.18 | 0.17 | 0.887 |
| Jack pine | **(Intercept)** | **2.38** | **0.15** | **16.12** | **<0.001** | -0.09 | 0.31 | -0.28 | 0.835 |
| N=292 | **Seed addition** | **1.14** | **0.08** | **15.02** | **<0.001** | **-2.06** | **0.37** | **-5.61** | **<0.001** |
|  | Mineral soil | -0.02 | 0.04 | -0.46 | 0.761 | 0.08 | 0.37 | 0.20 | 0.878 |
|  | **rSOL** | -0.25 | 0.14 | -1.84 | 0.138 | **0.86** | **0.23** | **3.76** | **<0.001** |
|  | **Pre-fire jack pine** | -0.17 | 0.13 | -1.26 | 0.350 | **-1.83** | **0.60** | **-3.02** | **0.008** |
|  | Canopy combustion | 0.09 | 0.13 | 0.68 | 0.668 | -0.46 | 0.20 | -2.29 | 0.051 |
|  | Exclosure | -0.04 | 0.07 | -0.60 | 0.688 | NA | NA | NA | NA |
|  | Stand age | NA | NA | NA | NA | 0.27 | 0.18 | 1.52 | 0.247 |
| Aspen | **(Intercept)** | **2.43** | **0.17** | **14.14** | **<0.001** | **1.24** | **0.30** | **4.19** | **<0.001** |
| N=330 | **Seed addition** | **0.51** | **0.11** | **4.61** | **<0.001** | -0.39 | 0.28 | -1.38 | 0.306 |
|  | **Mineral soil** | **-0.48** | **0.16** | **-2.94** | **0.008** | -0.23 | 0.33 | -0.70 | 0.668 |
|  | rSOL | -0.22 | 0.14 | -1.61 | 0.214 | 0.02 | 0.24 | 0.10 | 0.921 |
|  | Exclosure | 0.08 | 0.12 | 0.65 | 0.668 | NA | NA | NA | NA |
| Birch | **(Intercept)** | **2.64** | **0.30** | **8.69** | **<0.001** | -0.71 | 0.53 | -1.35 | 0.312 |
| N=168 | **Mineral soil** | **-0.76** | **0.14** | **-5.52** | **<0.001** | -1.20 | 1.76 | -0.68 | 0.668 |
|  | **rSOL** | **0.59** | **0.08** | **7.73** | **<0.001** | -0.17 | 0.21 | -0.83 | 0.636 |
|  | **Exclosure** | **0.88** | **0.07** | **12.53** | **<0.001** | NA | NA | NA | NA |
