# Supplementary material for: Material Legacies and Environmental Constraints Underlie Fire Resilience of a Dominant Boreal Forest Type
Source: Ecosystems. 2022 Jun 29;26(3):473–90. doi: 10.1007/s10021-022-00772-7 (PMC10167110; doi:10.1007/s10021-022-00772-7)
Supplement: Supplementary file 3 — Supplementary file3 (DOCX 12 kb) [file 10021_2022_772_MOESM3_ESM.docx]

ECOSYSTEMS MANUSCRIPT INFORMATION SHEET

MANUSCRIPT NUMBER: ECO-22-0007.R1

TITLE: Material legacies and environmental constraints underlie fire resilience of a dominant boreal forest type

AUTHORS: Day, Nicola; Johnstone, Jill; Reid, Kirsten; Cumming, Steven; Mack, Michelle; Turetsky, Merritt; Walker, Xanthe; Baltzer, Jennifer

CORRESPONDING AUTHOR:

Dr. Nicola Day

FAX:

PHONE:

EMAIL: [njday.ac@gmail.com](mailto:njday.ac@gmail.com)

RECEIVED 12-Jan-2022; ACCEPTED 07-May-2022

COLOR FIGURES: 4

COMMENTS:
